# Saving the sea cucumbers: Using population genomic tools to inform fishery and conservation management of the Fijian sandfish *Holothuria* (*Metriatyla*) *scabra*

**DOI:** 10.1371/journal.pone.0274245

**Published:** 2022-09-09

**Authors:** Kelly T. Brown, Paul C. Southgate, Chinthaka A. Hewavitharane, Monal M. Lal

**Affiliations:** 1 Discipline of Marine Studies, School of Agriculture, Geography, Environment, Ocean and Natural Sciences, The University of the South Pacific, Suva, Fiji; 2 School of Science, Technology and Engineering, and Australian Centre for Pacific Islands Research, University of the Sunshine Coast, Maroochydore, Queensland, Australia; National Cheng Kung University, TAIWAN

## Abstract

The sea cucumber *Holothuria (Metriatyla) scabra*, known as sandfish, is a high-value tropical echinoderm central to the global bêche-de-mer (BDM) trade. This species has been heavily exploited across its natural range, with overharvesting and ineffective fishery management leaving stocks in the Pacific region heavily depleted. In Fiji, sandfish stocks have not recovered since a 1988 harvest ban, with surveys reporting declining populations and recruitment failure. Therefore, to inform fishery management policy for the wild sandfish resource and to guide hatchery-based restocking efforts, a high-resolution genomic audit of Fijian populations was carried out. A total of 6,896 selectively-neutral and 186 putatively-adaptive genome-wide SNPs (DArTseq) together with an independent oceanographic particle dispersal model were used to investigate genetic structure, diversity, signatures of selection, relatedness and connectivity in six wild populations. Three genetically distinct populations were identified with shallow but significant differentiation (average *F*_st_ = 0.034, p≤0.05), comprising (1) Lakeba island (Lau archipelago), (2) Macuata (Vanua Levu), and (3) individuals from Yasawa, Ra, Serua island and Kadavu comprising the final unit. Small reductions in allelic diversity were observed in marginal populations in eastern Fiji (overall mean *A* = 1.956 vs. Lau, *A* = 1.912 and Macuata, *A* = 1.939). Signatures of putative local adaptation were also discovered in individuals from Lakeba island, suggesting that they be managed as a discrete unit. An isolation-by-distance model of genetic structure for Fijian sandfish is apparent, with population fragmentation occurring towards the east. Hatchery-based production of juveniles is promising for stock replenishment, however great care is required during broodstock source population selection and juvenile releases into source areas only. The successful use of genomic data here has the potential to be applied to other sea cucumber species in Fiji, and other regions involved in the global BDM trade. While preliminary insights into the genetic structure and connectivity of sandfish in Fiji have been obtained, further local, regional and distribution-wide investigations are required to better inform conservation efforts, wild stock management and hatchery-based restocking interventions for this valuable invertebrate.

## Introduction

The sandfish, *Holothuria* (*Metriatyla*) *scabra* is a high-value tropical sea cucumber central to the global bêche-de-mer (BDM) trade [1, 2]. National sea cucumber fisheries in the western Pacific have historically supported the greatest numbers of BDM exports, with volumes of 10,963, 8,719 and 6,081 tonnes recorded from PNG, Fiji and the Solomon Islands, respectively [3]. Unsurprisingly, the high fishing pressure experienced by stocks in the region has led to overfishing and fishery collapses for several species [4]. Today, many sea cucumber fisheries in the Pacific remain heavily exploited. These depleted stocks, with largely ineffective fishery management and inadequate regulatory measures [4], require immediate intervention to arrest stock collapses, and permit recovery.

Fiji, which until recently had remained one of two Pacific Island countries maintaining an open and lightly-regulated sea cucumber fishery [4, 5], implemented a complete ban on the harvest and export of all sea cucumbers in 2018 [6, 7], following a sandfish-specific ban in 1988 [8]. Currently, Fijian sea cucumber stocks are in a depleted state, with densities of all 20 commercially-traded species among the lowest recorded in the Pacific, with reference to healthy stocks in the region [5, 6, 9]. Furthermore, surveys of *H*. *scabra* population densities suggest that stock recovery has not occurred since the 1988 ban on harvests, with primarily sexually immature juveniles constituting populations at eight locations assessed [8].

It is evident that Fiji’s sea cucumber fishery requires additional regulatory measures to arrest declining wild stocks, with strict implementation and enforcement of quotas and collection size limits [8, 10]. These efforts could be reinforced by aquaculture and restocking efforts, to assist the recovery of wild populations [11, 12]. While previous research efforts have generated population demographic data on several commercially important sea cucumber species, which can be used for the development of minimum capture size guidelines and fishery quotas [5, 8, 13, 14], population genetic assessments remain lacking.

The integration of genetic data into sea cucumber stock assessments provides unique insights into stock health, including the amount of genetic diversity present in populations, the level of replenishment through gene flow from neighbouring stocks, as well as the presence of any local adaptation [15–17]. Genetic information can also directly inform aquaculture and restocking efforts involving *H*. *scabra* or other heavily depleted taxa, by identifying potential bottlenecks in the hatchery process where valuable genetic diversity is lost, ensuring that genetically diverse individuals are restocked in overfished areas [18–20].

Consequently, a high-resolution genomic audit of wild Fijian sandfish was completed, using a robust genotyping-by-sequencing approach (DArTseq) which had been previously evaluated for use in this species [21]. Insights from genomic data were combined with an independent oceanographic particle dispersal model to elucidate the genetic diversity, structure, connectivity, signatures of selection and relatedness of Fijian sandfish. Data generated will inform conservation efforts, wild stock management and hatchery-based restocking interventions for this valuable invertebrate.

## Materials and methods

### Sample collection and tissue sampling

**Fig 1:**
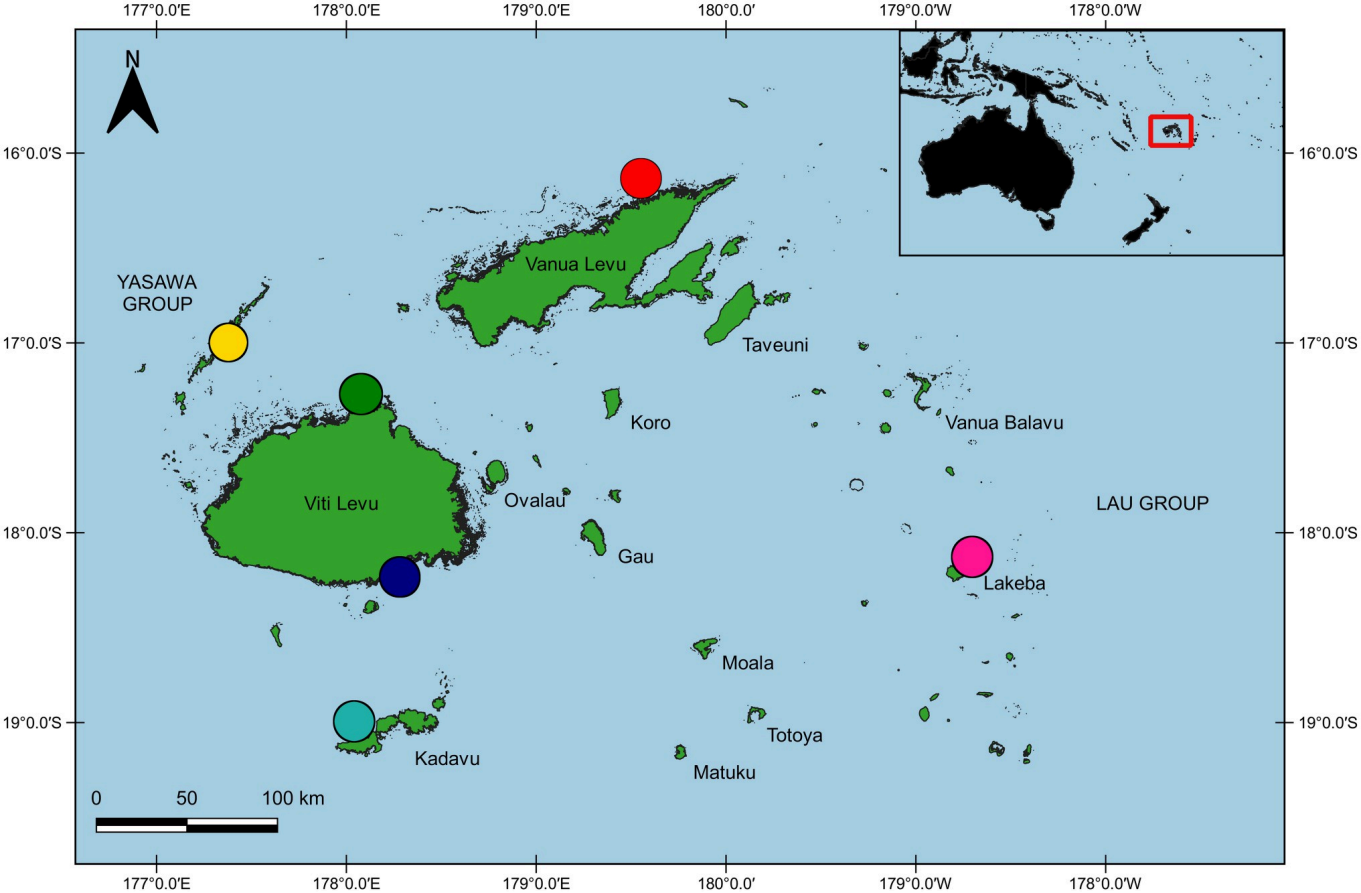
Map of sampling locations in Fiji where *Holothuria scabra* were collected, adapted from Lal et al. [22]. Dark shading along coastlines represents seagrass bed extents derived from Allen Coral Atlas data (https://allencoralatlas.org). Site codes are as follows: MA, Namuka district, Macuata Province, Vanua Levu; LA, Lakeba island, Lau island archipelago, Lau Province; KA, Tavuki district, Kadavu island, Kadavu Province; SA, Serua island, Serua Province; RA, Raviravi district, Ra Province, Viti Levu; YA, Nacula island, Yasawa island archipelago in the Ba Province. Produced using QGIS v 3.14.16-Pi and open source geographical data obtained from the SPC Pacific data hub (https://pacificdata.org/).

Adult *Holothuria scabra* were sampled from natural populations at six locations throughout Fiji (Fig 1): Namuka district, Macuata Province, Vanua Levu (n = 25, MA: 16° 11’ 59.89" S, 179° 41’ 15.74" E), Lakeba island, Lau island archipelago, Lau Province (n = 45, LA: 18° 10’ 58.58" S, 178° 45’ 02.15" E), Tavuki district, Kadavu island, Kadavu Province (n = 22, KA: 19° 04’ 29.46" S, 178° 06’ 43.32" E), Serua island, Serua Province (n = 42, SA: 18° 16’ 57.10" S, 177°55’ 57.03" E), Raviravi district, Ra Province, Viti Levu (n = 46, RA: 17° 24’ 11.63" S, 177° 59’ 55.03" E), and Nacula island, Yasawa island archipelago in the Ba Province (n = 31, YA: 16° 57’ 41.03" S, 177° 20’ 47.72" E). All sandfish were handled in accordance with the University of the Sunshine Coast’s animal ethics requirements and guidelines, in compliance with the Australian Code for the Care and Use of Animals for Scientific Purposes 8th Edition (2013). Written permission to collect sandfish specimens and sample tissues was obtained from the Fiji Ministry of Fisheries. A research permit to access coastal communities and engage in sandfish specimen collection was obtained from the Fiji Ministry of iTaukei Affiars (MTA-4/99/8-2).

Tissue samples measuring approximately 2.5 cm × 1.5 cm containing tube feet were collected non-destructively by removing a strip of skin from the ventrolateral anterior flanks of specimens. Tissues were preserved in 20% dimethyl sulfoxide (DMSO) salt solution [23], and maintained under refrigeration (4°C) until DNA extraction at the School of Agriculture, Geography, Environment, Ocean and Natural Sciences (SAGEONS), The University of the South Pacific in Suva, Fiji. Total genomic DNA was extracted using a modified CTAB:isoamyl alcohol protocol [21], and submitted for genotyping to Diversity Arrays Technology Ltd (DArT PL), in Canberra, Australia.

### DArTseq^™^ 1.0 library preparation and sequencing

Diversity Arrays Technology (DArT PL) proprietary genotyping by sequencing (DArTseq^™^) reduced-representation libraries were prepared as described by Kilian et al. [24] and Sansaloni et al. [25], with a number of modifications optimised for the sandfish genome as described by Lal et al. [21]. Briefly, genome complexity reduction was achieved with a double restriction digest, using a *Pst*I and *Sph*I methylation-sensitive restriction enzyme combination. Custom proprietary barcoded adapters (6–9 bp) were ligated to RE cut-site overhangs as described by Mercier et al. [26], with the adapters designed to modify RE cut sites following ligation.

Target "mixed" fragments [27] were selectively amplified using custom designed primers for each sample, under the following PCR conditions: initial denaturation at 94°C for 1 min, then 30 cycles of 94°C for 20 s, 58°C for 30 s and 72°C for 45 s, followed by a final extension step at 72°C for 7 min. Amplified samples were subsequently cleaned using a GenElute PCR Clean-up Kit (Sigma-Aldrich, cat.# NA1020-1KT). All samples were each normalised and pooled using an automated liquid handler (TECAN, Freedom EVO150), at equimolar ratios for sequencing on the Illumina HiSeq 2500 platform. After cluster generation and amplification (HiSeq SR Cluster Kit V4 cBOT, cat.# GD-401-4001), 77 bp single-end sequencing was performed at the DArT PL facility in Canberra, Australia.

### Marker filtering, genotype calling and filtering

Illumina CASAVA v.1.8.2 software was used for initial assessment of read quality, sequence representation and generation of FASTQ files from raw reads. Filtered FASTQ files were subsequently used for further filtering, variant calling and calling of final genotypes using the DArT PL proprietary software pipeline DArTtoolbox [28]. Within DArTtoolbox, the primary workflow first involved the package DArTsoft14 to remove reads with a quality score <25 from further processing and apply stringent filtering to the barcode region of all sequences to increase confidence in genomic region recovery.

Individual samples were then de-multiplexed by barcode, and subsequently aligned and matched to catalogued sequences in both NCBI GenBank and DArTdb custom databases to check for viral and bacterial contamination. Any matches for viral or bacterial sequences were removed from further processing. SNP and reference allele loci were identified in reduced-representation loci (RRL) clusters and designated scores of: "0" = reference allele homozygote, "1" = SNP allele homozygote and "2" = heterozygote, based on their frequency of occurrence. To ensure robust variant calling, all monomorphic clusters were removed, SNP loci had to be present in both allelic states (homozygous and heterozygous), and a genetic similarity matrix was produced using the first 10,000 SNPs called to assess technical replication error [29], and remove clusters containing tri-allelic or aberrant SNPs and overrepresented sequences.

Once SNP markers were identified, they were assessed for homozygote and heterozygote call rate, frequency, polymorphic information content (PIC), average SNP count, read depth and repeatability using DArT PL’s KD Compute package. Upon receiving the raw genotype dataset from DArT PL, it was further filtered, using a nested criteria of call rate and average polymorphic information content (PIC, highest to lowest rankings for both criteria), to retain only a single, highly informative SNP at each genomic locus for population genomic analyses. The dataset was further filtered for global call rate (95%), read depth (>8), average PIC (1%) MAF (2% per population) and average repeatability (95%). All loci were assessed for departure from Hardy-Weinberg Equilibrium (HWE) using Arlequin v.3.5.1.3 [30], using an exact test with 10,000 steps in the Markov Chain and 100,000 dememorisations.

Identification of loci under potential selection was achieved using two software packages, BayeScan v.2.1 [31, 32] and HacDivSel [33], independently. Bayescan computations were carried out at 0.01, 0.05, 0.1, 0.2 and 0.5 False Discovery Rate (FDR) thresholds (see [21] for further details). HacDivSel employs a different approach to BayeScan, in that populations are submitted pairwise for detection of divergent selection. The extreme-outlier (EOS) test was used, where an algorithm searches for different outlier clusters and then performs a conditional LK test where more than one cluster is found based on the magnitude of *F*_st_ values. Putative *F*_ST_ outlier loci identified during both runs were removed and segregated into a separate outlier marker dataset, and all remaining SNPs tested for departure from Hardy-Weinberg Equilibrium (HWE) in Arlequin v.3.5.1.3 [30], using an exact test with 10,000 steps in the Markov Chain and 100,000 dememorisations. Loci under selection were identified and removed, and a final dataset containing selectively neutral loci was produced. For putatively adaptive loci, BLAST searches for SNP flanking sequences and any potential functional annotations were also carried out.

### Assessment of genomic diversity and population differentiation

For assessment of genomic diversity within and between sample groups on selectively neutral markers, allelic diversity indices including the average observed (*H*_o_) and average expected heterozygosities corrected for population sample size (*H*_n.b._), were computed using Genetix v.4.05.2 [34]. Genetix was also used to calculate Wright’s inbreeding coefficients (*F*_is_) per sample group and mean numbers of alleles per locus (*A*, MAF ≥ 5%). The number of private alleles (*A*_p_, at MAF ≥5%) was computed with HP-RARE v.1.0 using the rarefaction method [35], and verified with the *R* package PopGenKit [36], while the average multi-locus heterozygosity (MLH) per population was determined after [37]. Effective population size based on the linkage disequilibrium method (*N*_*eLD*_) was estimated for each population, using NeEstimator v.2.01 [10].

### Resolution of genetic structure at selectively neutral and putatively adaptive loci

Pairwise *F*_st_ estimates for each sample group were calculated using Arlequin v.3.5.1.3 with 10,000 permutations [30], together with Nei’s standard genetic distances (*D*_S_,1978) in Genetix v.4.05.2 [34]. A Discriminant Analysis of Principal Components (DAPC) was employed to examine broad genetic structure in the *R* package *adegenet* 1.4.2 [38–40]. The DAPC was carried out for both selectively-neutral and putatively adaptive loci, and an α-score optimisation used to inform the number of principal components to retain. Additionally, the ‘find.clusters’ function of *adegenet* determined the optimal number of actual clusters using a Bayesian Information Criterion (BIC) method.

For estimation of proportional ancestral contributions and population stratification among sample groups (selectively neutral loci), the ADMIXTURE package was used [41, 42]. ADMIXTURE employs the likelihood model utilised in the STRUCTURE analysis package [43]; however, instead of adopting a Bayesian approach and a Markov chain Monte Carlo (MCMC) algorithm to sample posterior likelihood distributions, it applies a Maximum Likelihood method to estimate parameters [41]. Assessment of the optimal k threshold was carried out by examining ADMIXTURE’s cross-validation (cv) error statistic, by specifying the—cv flag during computations. Additionally, family relationships among individuals within sample groups were assessed using ML-RELATE [44] to identify any parent-offspring, full or half-sib pairs present.

To investigate fine-scale stratification at selectively neutral loci, network analyses were also carried out using the Netview R package [45, 46]. Netview R population networks were generated based on a shared allele 1-identity-by-state (IBS) distance matrix created in the PLINK v.1.07 toolset [47]. Each network was constructed following computation of the maximum number of nearest neighbours for a given individual [45, 46, 48]. Individual networks are then visualised and edited in the Cytoscape v.2.8.3 network construction package [49]. The IBS matrices and corresponding networks were constructed at various thresholds of the maximum number of nearest neighbour (mk-NN) values between 1 and 50, after which the optimal network for representation was selected based on cluster stability [45]. For assessment of hierarchical genetic structure, an analysis of molecular variance (AMOVA) was computed in GenAlEx v6.502 fitting populations as sources of variation [50]. Testing of an isolation-by-distance (IBD) model of genetic structure was also implemented in GenAlEx v6.502 with a Mantel Test utilising 999 permutations to assess statistical significance.

### Hydrodynamic particle dispersal modelling

#### Model design and simulation approach

The particle dispersal modelling software DisperGPU (https://github.com/CyprienBosserelle/DisperGPU), was used to examine potential larval transport pathways between source (sandfish sampling locations) and sink areas. The approach described by Lal et al. [15] for the black-lip pearl oyster was adopted and modified for *H*. *scabra*. Briefly, the analysis utilised two models: the DisperGPU particle model which simulated dispersal, and the HYbrid Coordinate Ocean Model (HYCOM) which provided hindcast current velocity data and hydrodynamic forcing to drive the former. Larvae were simulated as particles seeded into defined polygons seagrass bed area extents for each sampling location extracted from Allen Coral Atlas data (https://allencoralatlas.org). During simulations, particle positions were tracked over a fixed period of time to emulate the Pelagic Larval Duration (PLD) of *H*. *scabra*.

DisperGPU utilises a standard Lagrangian formulation, taking into account particle position (latitude and longitude), displacement during model time steps (set as one day), eddy diffusivity and the surface current speed at the location of the particle [27, 51]. The HYCOM model has a resolution (*dx*) of 1/12^th^ of a degree and is output every day [52, 53]. Further information on model formulation is described in [15, 54]. To generate simulations, gridded hindcast surface current data from the HYCOM model were interpolated into dispersal steps, after which current velocities at each particle position were calculated through bi-linear interpolation. Particle positions during each model time step (1 day) were recorded and particle ages were retained and increased during each step.

#### *H*. *scabra* reproductive biology, model configuration and dispersal visualisation

The occurrence and extent of seagrass beds in Fiji were obtained from an Allen Coral Atlas (https://allencoralatlas.org) benthic cover dataset layer file, and separated from other benthic class layer objects (e.g. coral/algae, rock, rubble etc.) using QGIS v 3.14.16-Pi. To generate a seed file as input for DisperGPU, a seagrass habitat point layer was acquired by first extracting vertices from the seagrass polygon layer file described earlier and adding coordinate points as a geometry attribute in QGIS. This resulted in 3,159,114 feature points being captured. For each sampling site, a polygon was extracted to account for seagrass bed area extent where specimens were collected and imported to DisperGPU as discrete seed files.

Sandfish are simultaneous broadcast spawners with larvae developing over a PLD of 25 days and mature females produce between 9–17 million eggs depending on their size and age [55, 56]. Larvae have limited swimming capability [55] and disperse via prevailing surface ocean currents. Wild sandfish spawn year round, however a period of peak spawning has been observed in the southwest Pacific from October-December [9]. In Fiji, this peak occurs in November [6]. These biological attributes were used to set the following particle model parameters: simulation duration of 30 days (PLD of 25 days plus a "settlement and recruitment" window of 5 days) and simulations run in November to account for the peak spawning season in Fiji.

Particles were seeded everyday for the first 3 days; no particle mortality or competency was simulated and a uniform distribution of adult sandfish within seagrass beds was assumed. A fixed number of 256,000 particles uniformly distributed across all seed area polygons was seeded each day over 3 days, making a total of 4,608,000 particles released during simulations for each respective spawning season. This quantity of particles was selected based on constraints on available computational power, and DisperGPU input requirements (see https://github.com/CyprienBosserelle/DisperGPU for further information). The El Niño Southern Oscillation (ENSO) system is known to influence surface ocean current dynamics in Fiji [15], and therefore two dispersal simulations were run to explore differences between representative El Niño (2012) and La Niña (2014) years.

Particle positions were extracted each day from the start of each simulation run and plotted on a map using the GMT software package [57]. These plots were annotated with model time step (day) and assembled to produce animations of each run (see S1 and S2 Figs), which permitted visualisation of particle movement and potential transport pathways.

## Results

### DArTseq genotyping and marker filtering

The raw dataset contained a total of 49,051 SNPs genotyped across 211 individuals. The removal of duplicate (clone) SNPs, resulted in 36,574 SNPs remaining (25.4% reduction), while further filtering for call rate (95%), read depth (>8), average PIC (1%) MAF (2% per population) and average repeatability (95%), left 7,082 SNPs remaining in the dataset. Screening for *F*_st_ outliers identified 186 SNPs, comprising HacDivSel extreme outliers (n = 157), and Bayescan 2.1 outliers at an FDR = 0.01 (n = 29), resulting in the retention of 6,896 selectively neutral genome-wide SNPs. A separate dataset of 186 putatively adaptive SNPs was retained for investigation of selective signatures in genetic structure. No loci were found to deviate from HWE across the six populations, and subsequently no loci were removed from the dataset. A total of 6,896 SNPs remained in the final filtered selectively-neutral dataset, and were used for performing population genomic analyses.

### Genomic diversity

Effective population sizes (*N*_*eLD*_) varied by geographic location (see Table 1, numbers in square brackets indicate 95% CI). The largest *N*_*eLD*_ were detected in three populations on or in close proximity to the island of Viti Levu, the largest island in Fiji. These were Ra (3839.3; [3235.8–4718.4]), Nacula island, Yasawa (1947.3; [1693.7–2292.6]) and Macuata (988.3; [890.0–1110.9]). The smallest *N*_*eLD*_ were observed in sandfish sampled from the more isolated islands of Lakeba, Lau (43.6; [43.5–43.8]) and Kadavu (67.9; [67.3–68.6]).

**Table 1:** Genetic diversity indices and relatedness computed for the *Holothuria scabra* populations sampled. Parameters calculated include the effective population size by the linkage disequilibrium method (*N*_*eLD*_; 95% confidence intervals indicated within brackets), mean number of alleles per locus (*A*), standardised private allelic richness (*Ap*, MAF ≥5%): The total number of loci with private alleles detected per population is shown in bold, effective number of alleles (N_eff_), number of locally common alleles (MAF ≥5%) found in <50% of all 6 populations tested, percentage of polymorphic loci, observed heterozygosity (*H*_o_), average expected heterozygosity corrected for population sample size (*H*_n.b._), inbreeding coefficients (*F*_is_) and average individual multi-locus heterozygosity (MLH). All computations were generated using a dataset containing 6,896 genome-wide SNPs.

Population	n	*N*_*eLD*_ [95% C.I.]	*A* (≥5%)	*Ap* (≥5%) (± SD)	N_eff_ (± SD)	Locally common alleles (≥5%)	% polymorphicloci	*H*_*o*_(± SE)	*H*_n.b._ (± SE)	*F*_is_ (p<0.01)	Av. MLH (± SD)
Tavuki, Kadavu KA	22	67.9 [67.3–68.6]	1.954	0.000±0.000	1.455±0.004	0.113±0.004	95.4	0.265±0.002	0.284±0.002	0.067	0.265±0.009
Lakeba, Lau LA	45	43.6 [43.5–43.8]	1.912	0.001±0.000 **(3)**	1.435±0.004	0.095±0.004	91.2	0.254±0.002	0.266±0.002	0.047	0.254±0.006
Macuata, Vanua Levu MA	25	988.3 [890.0–1110.9]	1.939	0.000±0.000	1.452±0.004	0.105±0.004	93.9	0.260±0.002	0.280±0.002	0.073	0.251±0.05
Ra, Viti Levu RA	46	3839.3 [3235.8–4718.4]	1.985	0.000±0.000	1.456±0.004	0.121±0.004	98.5	0.262±0.002	0.281±0.002	0.068	0.262±0.009
Serua island SA	42	507.2 [491.7–523.7]	1.982	0.000±0.000	1.461±0.004	0.121±0.004	98.2	0.238±0.002	0.284±0.002	0.163	0.226±0.072
Nacula island, Yasawa YA	31	1948.3 [1693.7–2292.6]	1.968	0.000±0.000	1.454±0.004	0.116±0.004	96.8	0.265±0.002	0.281±0.002	0.058	0.265±0.006

Mean numbers of alleles per locus (*A*) were comparable among all populations (*A =* 1.956 ± 0.0114 SE), varying from *A* = 1.912 at Lakeba island to *A* = 1.968 at Nacula island, Yasawa. Lakeba island was also the only population containing private alleles (*Ap* = 0.001, 3 private alleles). The effective number of alleles were similar among all sample groups (N_eff_ range of 1.452–1.461), with the exception of Lakeba island where N_eff_ = 1.435. Similar trends were evident in the numbers of locally common alleles and the percentage of polymorphic loci, suggesting a small reduction in diversity at Lakeba island and Macuata.

Patterns in observed and expected heterozygosities did not differ markedly for all sample groups, with the exception of Serua island, where *H*_o_ = 0.238, *H*_n.b._ = 0.284 (see Table 1). Serua island is a source of broodstock for the Fiji Ministry of Fisheries sandfish hatchery, and also a restocking location where hatchery produced juveniles are released, possibly accounting for this observation. Inbreeding coefficient values were similar for Kadavu, Macuata, Ra and Yasawa sandfish (range of 0.058–0.068), with Lau specimens producing the lowest value (F_is_ = 0.047, p<0.01). Serua island individuals, however, exhibited an over two-fold difference, with a value of 0.163. Trends in multi-locus heterozygosity (MLH) were similar, with the lowest value of 0.226±0.072 obtained for Serua island. Considered together, these data highlight a small but measurable heterozygote deficit in Serua island sandfish, with small reductions in allelic diversity among Lakeba Island and Macuata populations.

### Genetic structure and relatedness

Pairwise *F*_st_ estimates were shallow but significant for most comparisons (Table 2) with the exception of Ra with Serua island individuals. Due to geographic isolation from all other sample groups, sandfish from Lakeba island showed the highest level of differentiation (pairwise *F*_st_ range of 0.055–0.063, cf. 0.005–0.019), followed by individuals sampled from Macuata, Vanua Levu (*F*_st_ range of 0.014–0.019). Similar patterns were reflected in standard genetic distance estimates, with Lakeba island sandfish being the most divergent group (Nei’s *D*_S_ = 0.023–0.025, cf. 0.001–0.008 for other pairwise comparisons). Results of the Mantel test revealed no significant relationship between genetic and geographic distances among populations (r = 0.037, p = 0.23, 999 permutations), and partitioning of genetic variance by AMOVA reported that variation within (78%) and among (19%) individuals accounted for most of the variability in the dataset, with the smallest proportion (3%) originating between populations.

**Table 2:** Pairwise population differentiation estimates computed for *Holothuria scabra* sampled using 6,896 SNPs. Pairwise *F*_st_ values (Weir and Cockerham’s 1984 unbiased method) are reported below the diagonal, and were generated in Arlequin v3.5.1.3 following 1,000 permutations. Nei’s (1978) standard genetic distances (*D*_S_) are reported above the diagonal and were computed in Genetix v4.05.2 with 10,000 permutations.

	Tavuki, Kadavu KA	Lakeba, Lau LA	Macuata, Vanua Levu MA	Ra, Viti Levu RA	Serua Viti Levu SA	Nacula, Yasawa YA
Tavuki, Kadavu KA	-	0.023	0.006	0.002	0.001	0.002
Lakeba, Lau LA	0.058*	-	0.025	0.024	0.023	0.025
Macuata, Vanua Levu MA	0.014*	0.063*	-	0.008	0.007	0.007
Ra, Viti Levu RA	0.005*	0.060*	0.019*	-	0.001	0.001
Serua Viti Levu SA	0.000	0.055*	0.016*	0.000	-	0.001
Nacula, Yasawa YA	0.005*	0.061*	0.017*	0.001	0.000	-

**Fig 2:**
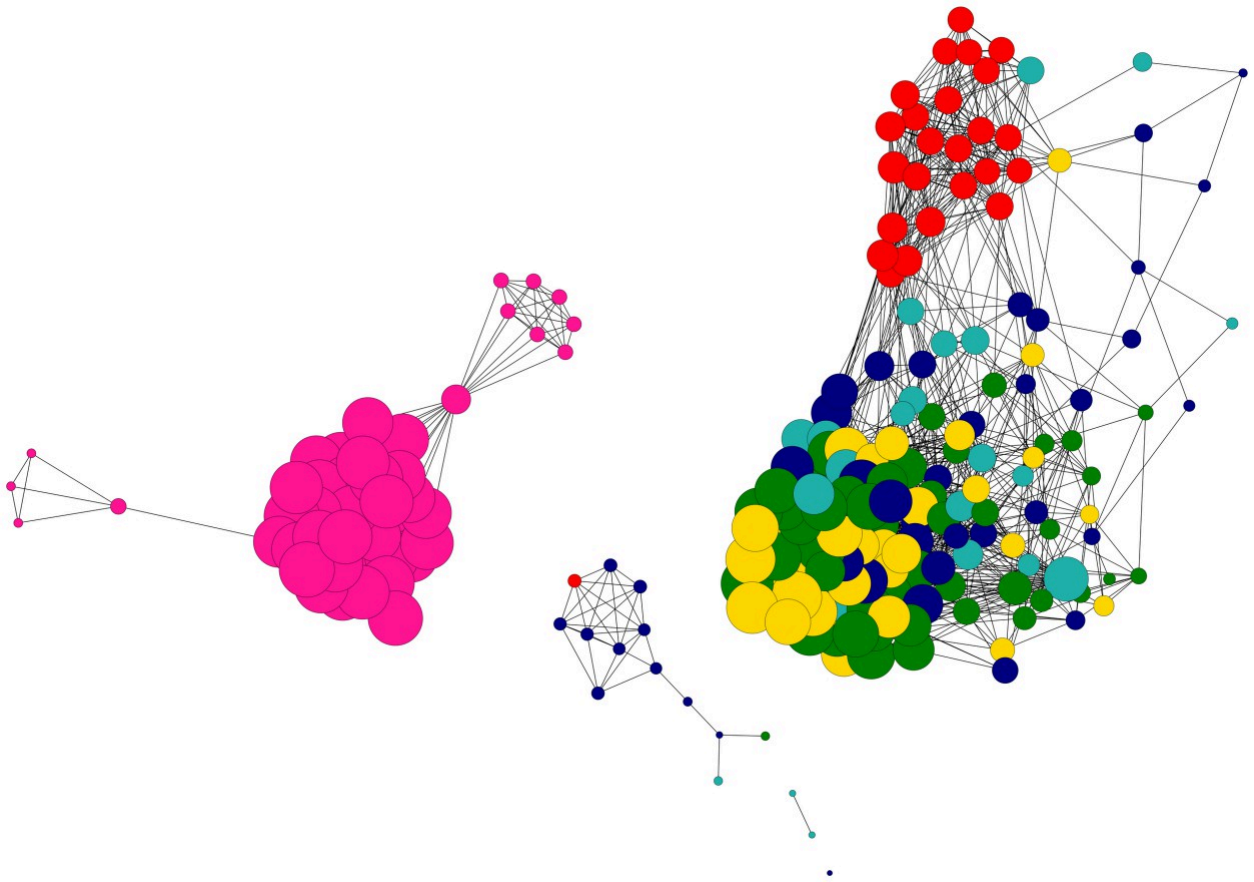
High resolution population network of Fijian *Holothuria scabra* generated using Netview R [45]. The network has been visualised at a maximum number of nearest neighbour (k-NN) threshold of 30, using 6,896 SNPs and 211 individuals. Each dot represents a single individual, and population colours correspond with Fig 1. Node sizes have been mapped to the relatedness (neighbourhood connectivity based on IBS distances) of individual animals, and the network constructed using the organic topology framework option in Cytoscape v.2.8.3.

**Fig 3:**
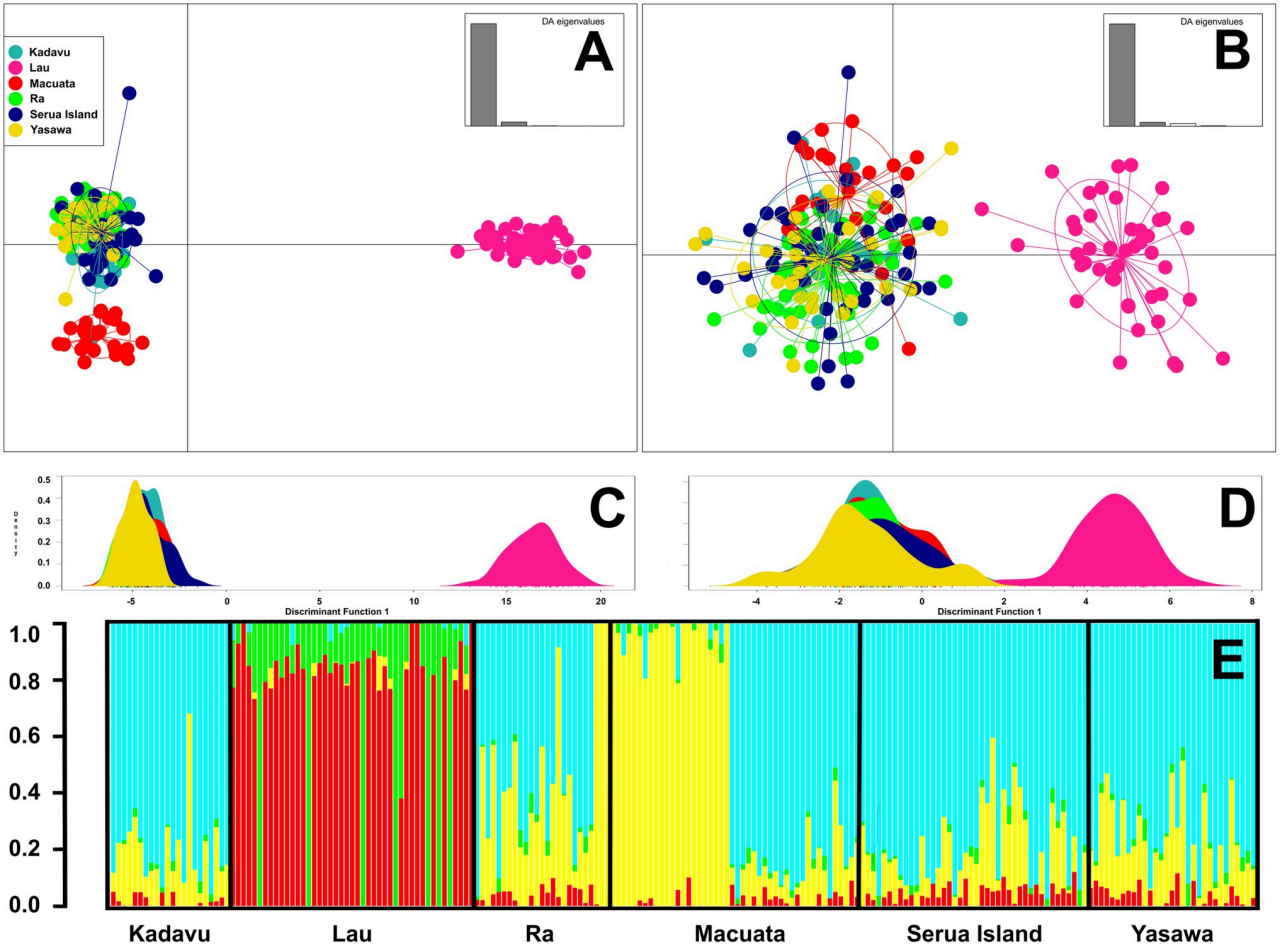
Visualisation of genetic structure in Fijian *Holothuria scabra* generated using Discriminant Analysis of Principal Components (DAPC) scatterplots and ADMIXTURE barplot. **A**: Alpha-score optimised DAPC scatterplot with 11 DFs (6896 selectively neutral SNPs) and **B**: Alpha-score optimised DAPC with 7 DFs (186 putatively adaptive SNPs). **C** and **D** depict DAPC plots generated using a single discriminant factor for the 6896 selectively neutral and 186 putatively adaptive SNPs, respectively. **E** shows an ADMIXTURE barplot of proportional ancestral contributions at a k = 4 threshold.

Examination of fine-scale selectively neutral genetic structure among all sample groups with Netview R were concordant with pairwise *F*_st_ and genetic distance estimates, resolving two primary clusters of individuals (Fig 2). Sandfish sampled at Lakeba island segregated within their own discrete and compact cluster, whereas almost all remaining individuals grouped into a large diffuse group containing individuals from Kadavu, Ra, Serua island, Yasawa and Macuata. Individuals from Macuata formed their own compact cluster within this larger grouping, reflecting their relative geographic isolation from other sites. Estimation of proportional ancestral contributions using the ADMIXTURE package indicated stratification of all individuals sampled into three groups at k thresholds ≥ k = 4 (Fig 3E). These groups comprised Lakeba island and Macuata separately, with all other individuals aggregated into the third group.

Resolution of genetic structure was also performed with a DAPC, with both selectively neutral and putatively adaptive marker datasets used. At neutral markers, the DAPC scatterplot (Fig 3A) produced a very similar pattern to that obtained with Netview R, with differentiation of Macuata and Lakeba island individuals. Interestingly at putatively adaptive loci, two clusters were resolved in the DAPC (Fig 3B). The first large cluster contained sandfish sampled at Ra, Kadavu, Yasawa, Serua island and Macuata, while the second contained only individuals from Lakeba island. These results suggest local adaptation in the presence of gene flow, perhaps due to habitat heterogeneity. Given the relatively small spatial scale of sampling (up to ~300 Km) between the furthest sites, these data are important considerations for fishery and translocation management of wild *H*. *scabra*. BLAST searches for functional annotations using flanking sequences associated with the *F*_st_ outlier SNPs unfortunately did not return any informative results.

An examination of individual pairwise relatedness and kinship within each sample group using MLRelate revealed an absence of any parent-offspring, full-sib or half-sib relationships among Macuata, Ra, Serua island or Yasawa sandfish (Table 3), suggesting unrelated individuals were sampled. Among Lakeba island individuals however, 27 full-sib and 22 half-sib relationships were detected, possibly reflective of a smaller, isolated population where close relatives were sampled.

**Table 3:** Summary of individual pairwise relationships assessed using MLRelate for each sample group. The number and mean ± SD of the natural log likelihood of each type of relationship (in brackets) are presented.

Relationship	Kadavu	Lau	Macuata	Ra	Serua Is.	Yasawa
**# relationships tested**	231	990	300	1035	861	465
**Parent/Offspring**	0	0	0	0	0	0
**Full sib**	1 (-2806)	27 (-3437 ± 127)	0	0	0	0
**Half sib**	0	22 (-3763 ± 166)	0	0	0	0
**Unrelated**	230 (-4109 ± 120)	941 (-3883 ± 105)	300 (-3777 ± 929)	1035 (-4147 ± 46)	861 (-3906 ± 641)	465 (-4105 ± 56)

* Significant values p≤0.05

#### Particle dispersal modelling

**Fig 4:**
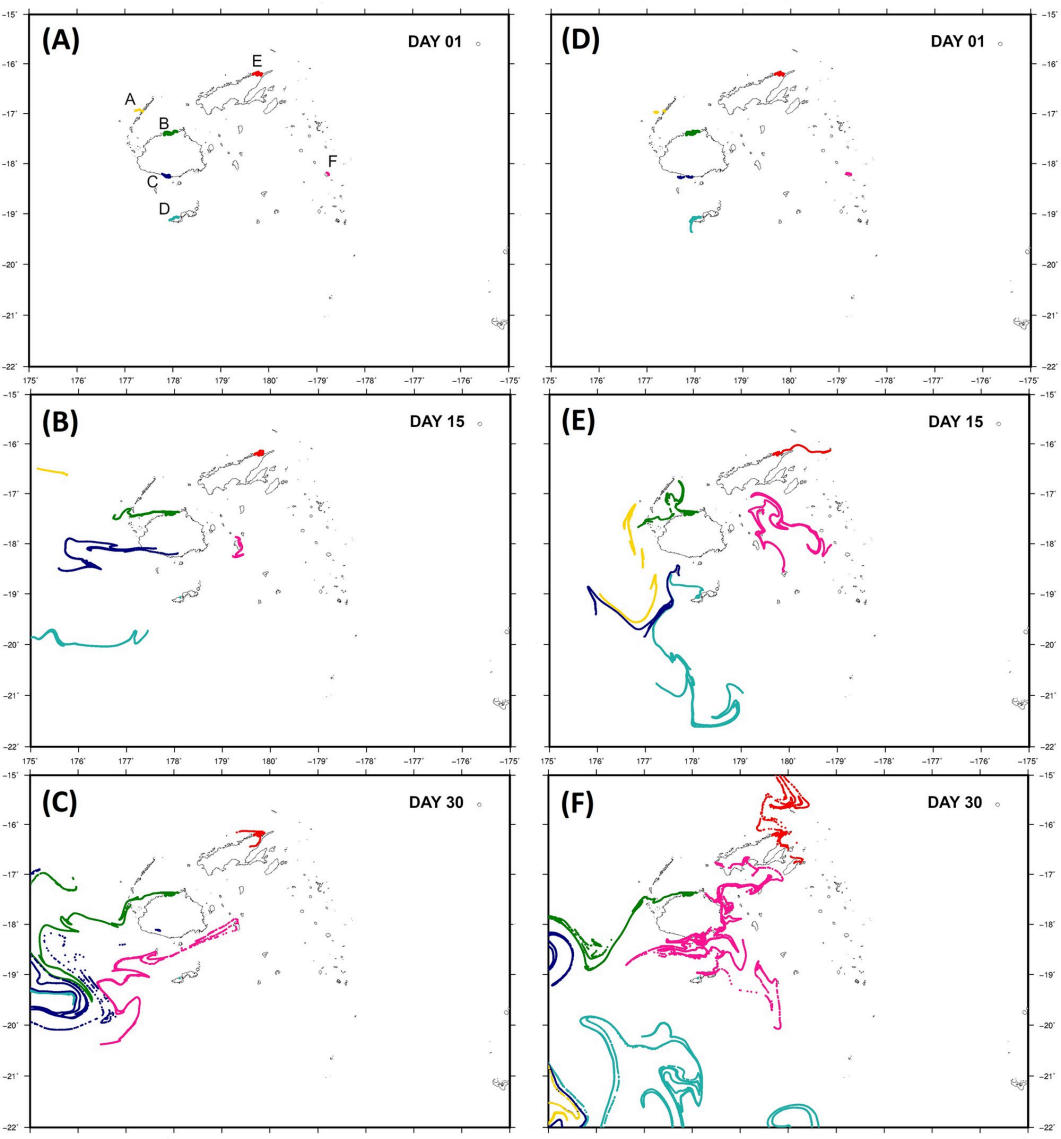
Particle dispersal simulation results for a La Niña (2012: A-C) and El Niño (2014: D-F) HYCOM datasets. Dispersal simulation progressions are shown for days 1, 15 and 30 for each year respectively. Animations of these simulations are available in S1 and S2 Figs. Base maps for dispersal visualization are adapted from Lal et al. [22]. Letters denote the following sampling locations: LA, Lakeba island, Lau archipelago; YA, Nacula island, Yasawa archipelago; SA, Serua island, Serua Province, Viti Levu; MA, Namuka district, Macuata Province, Vanua Levu; KA, Tavuki district, Kadavu, and RA, Raviravi district, Ra Province.

Hydrodynamic particle dispersal simulations showed highly directional current-driven patterns over Fiji, with different fluxes between El Niño and La Niña year datasets (Fig 4 and S1 and S2 Figs). Particle admixture was restricted in both datasets, with mixing only occurring in turbulent flows over deeper water some distance away from major coastlines. These patterns agree well with patterns of reduced connectivity observed in the genetic data for the Lau and Macuata sampling sites, and moderate connectivity between locations in eastern Viti Levu.

During both simulations, particles were advected predominantly westward, likely due to the east-to-west flow of the South Equatorial Current (SEC) which influences surface water movement in Fiji. For the El Niño year simulation, dispersal was strongly directional, with admixture only evident during eddy effects in south-western Viti Levu over deeper water towards the end of the simulation (day 24 onwards, see Fig 4(A)–4(C)). Particle movement differed during the La Niña year simulation, with an earlier onset of eddy effects and oscillation of particle groups. Initial particle flux patterns were westward, however, by day 10 circular surface water movements resulted in earlier admixture (see Fig 4(D)–4(F)). Particles seeded in the Lau sampling site reached and diffused across the eastern Viti Levu coastline (by day 23), Macuata particles dispersed to the north while particles seeded in Ra, Yasawa, Serua island and Kadavu merged off southwestern Viti Levu.

## Discussion

We report for the first time a high-resolution, country-wide genetic audit of wild sandfish populations in Fiji, and identify patterns of both selectively-neutral and putatively adaptive genetic structure, connectivity, diversity and relatedness. These data will be instrumental for informing fishery management of wild sandfish in Fiji, and will also direct conservation and restocking efforts for depleted populations. Given the history of heavy exploitation of sandfish and other sea cucumbers in Fiji and more broadly the south Pacific region, insights gained through this study may be applied to other taxa and in other locations for sustainable exploitation of this valuable but still poorly understood resource.

### Genetic structure, connectivity and gene flow

Three genetically distinct populations were identified at selectively-neutral genome-wide SNP markers for sandfish sampled from six locations in Fiji, with Lakeba island (Lau archipelago) in the east, Macuata in the north, and the remaining individuals from Yasawa, Ra, Serua island and Kadavu constituting the final group. These data suggest an isolation-by-distance model of dispersal operating in Fiji with population fragmentation occurring towards the east, although both an AMOVA and Mantel test were not able to detect a significant IBD pattern. Similar partitioning of genetic variance to within individuals rather than populations was detected by Nowland et al. [58] for northern Australian and Papua New Guinean sandfish. Examination of genetic structure at putatively adaptive loci however presents evidence for shallow IBD-mediated structure, with only sandfish from Lakeba island segregating in a discrete cluster from all other sample groups at 186 *F*_st_ outlier SNPs (Figs 2 and 3). Ravago-Gotanco and Kim [59] similarly did not detect IBD patterns when all 15 populations in their study from the Philippines were considered, however, their most divergent population pair from the Sulu Sea were more differentiated relative to all other pairwise comparisons over the same geographic range of 75–1,500 Km.

A parallel pattern of genetic structure in Fiji was observed by López et al. [8] who used CO1 data for the sea cucumber lollyfish (*Holothuria atra*), a congener of sandfish which is found in similar habitats. These authors reported net gene flow from east to west, with high differentiation between an island (Taveuni) and mainland (Vanua Levu) populations of *H*. *atra*. Hydrodynamic particle dispersal modelling implemented in this study to verify patterns of genetic structure and connectivity also support these conclusions, with highly directional westward transport pathways, mediated by the South Equatorial Current over both El Niño and La Niña event year simulations (Fig 4). The geographic extent of Fiji is relatively small, with up to a ~600 km straight line distance from the Yasawa archipelago in the west to the Lau archipelago at the eastern limit of the country. While areas of suitable contiguous habitat for sandfish (i.e., seagrass beds and sandy/muddy substrates) are widespread [60], they are separated by stretches of deep water, particularly towards island archipelagos. When coupled with prevailing ocean current-mediated larval dispersal, it is likely that populations inhabiting Fiji’s outer island groups may be functionally weaker larval sinks as a result of reduced gene flow and habitat availability, relative to more central populations.

Population genetic structure of *H*. *scabra* has been examined previously using various marker types including allozymes [61, 62], the mitochondrial CO1 gene [63], and microsatellites [58, 59, 64] at various sites within its Indo-west Pacific distribution. All studies have identified discrete genetic units in northern Australia and the Solomon Islands [61]; New Caledonia, northern Australia and the Solomon Islands [62]; Darwin and Cape York in northern Australia with Papua New Guinea [58]; and the Philippines [59]. Apart from a preliminary study to assess the efficacy of genome-wide genotyping in sandfish [21], the current study is the first to apply genome-wide SNPs to perform a high-resolution genetic audit of wild populations. From data presented here and with insights from the aforementioned studies, it is evident that detectable genetic structure is commonplace in wild sandfish, governed by the extent of gene flow connecting populations and the availability of suitable habitat. These findings differ from studies of other broadcast spawning benthic invertebrates such as pearl oysters [15, 16] and spiny lobsters [65], where greater levels of panmixia are observed.

In the Philippines, Ravago-Gotanco and Kim [59] reported the highest levels of genetic differentiation were observed when sandfish populations from peripheral biogeographic regions were compared against central regions. Similar patterns were reported by Nowland et al. [58] between Northern Australia and Papua New Guinea at maximum distances of ~4,000 km, and Northern Australia and the Solomon Islands at distances of up to ~2,000 km [61]. As peripheral populations become separated by stretches of open ocean with fewer islands providing suitable habitat for recruitment, genetic differentiation and population fragmentation increases, fitting the Core-Periphery Hypothesis (CPH) model of genetic variation and structure. The CPH model theorises that within a species’ distribution over a geographical gradient, genetic diversity is highest around the core region, with populations becoming smaller, fragmented and divergent towards the periphery where conditions for persistence are less than ideal [66–68].

A further factor compounding divergence and population fragmentation in sandfish specifically relates to the species’ life history, as development occurs through a highly dispersive planktonic larval stage [69], before requiring initial settlement on seagrass leaves with later migration to sand substrates [70, 71]. This biphasic settlement process [26] likely has a strong influence on the colonisation success of remote and/or new regions, as successful recruitment is reliant on the presence of seagrass.

### Genomic diversity and relatedness

The natural distribution of *H*. *scabra* is extensive within the tropical Indo-Pacific, however in the Pacific basin, Fiji represents the easternmost extent of its range [72]. This positions Fijian populations at the periphery of the known natural distribution, away from core populations (as per the CPH model) in the coral triangle region, encompassing Indonesia, Malaysia, the Philippines, Papua New Guinea and the Solomon Islands [26]. Ravago-Gotanco and Kim [59] reported that Philippine sandfish populations exhibited greater genetic diversity, allelic richness and observed heterozygosities relative to Papua New Guinean and Northern Australian populations. Similar observations have been made for other benthic invertebrate taxa, including the silver-lip pearl oyster *Pinctada maxima* [73] and mud crab *Scylla olivacea* [74].

Higher levels of genetic diversity within core relative to peripheral populations under the CPH model are expected, as the availability of suitable habitat is higher, population sizes are larger and stronger connectivity between populations is maintained. Conversely, selection pressures are intensified, connectivity is weaker and consequently smaller, fragmented and less diverse populations exist within peripheral habitats [66]. Distribution of genomic diversity varied between sampling locations in Fiji, with smaller effective population sizes and lower allelic diversity observed in the more isolated populations towards the east of the country, e.g., the islands of Kadavu and Lakeba, consistent with CPH predictions. These data suggest the presence of a diversity gradient from east to west, concordant with observations of genetic structure and pattern of gene flow.

Small heterozygote deficits were evident in individuals sampled at Lakeba island and Macuata, which are marginal populations within Fiji. Within the Philippine archipelago, Ravago-Gotanco and Kim [59] made similar observations for 15 sandfish populations separated by central vs. peripheral marine biogeographic regions. At Serua island in Fiji, individuals exhibited lower observed and multi-locus heterozygosity and a higher inbreeding coefficient relative to other populations. This observation may be the result of a sandfish restocking programme operated by the Fiji Ministry of Fisheries, where broodstock are sourced from Serua island and resulting hatchery produced juveniles are released in the same area.

Among the other Fijian populations sampled, no signatures of genetic bottlenecks were detected, perhaps due to the overriding effects of gene flow [75]. This observation is somewhat surprising, given the history of heavy exploitation of sandfish and other sea cucumber taxa. Historically, the sea cucumber fishery in Fiji has been exploited since 1813 [5], with a 2013 survey of populations of 20 species (including *H*. *scabra*) in the Lau archipelago reporting that the fishery there is in severe decline to collapse, with population densities generally below the suggested within-species limit of 10–15 individuals ha^-1^ required to avoid depensation [76]. These levels of sustained overharvesting in the Lau archipelago combined with their geographic position as marginal populations likely explain the reductions in genetic diversity observed at Lakeba island, together with the detection of 27 full-sib and 22 half-sib relationships among individuals from that population (Table 3). Being a smaller, isolated population, it is plausible that closely related individuals were sampled, in contrast to the other Fijian populations which were all unrelated.

### Implications for fishery and restocking management in Fiji

#### Management of the wild resource

The wild sea cucumber resource in Fiji has been assessed using traditional fishery census-based methods in several studies [4, 5, 8, 76, 77], and all suggest species richness and densities at the majority of sites surveyed are among the lowest recorded compared to reference densities for healthy stocks in the Pacific region [5, 8]. A total of 27 species of sea cucumber are present in Fiji, of which 20 species (including *H*. *scabra*) are exploited in the commercial bêche-de-mer trade [5]. The most recent stock assessment effort reported that as stocks of high-value and shallow water species such as *H*. *scabra* are depleted, fishing effort has shifted towards low and medium-value species such as *H*. *atra* [78].

For *H*. *scabra*, it is evident that current fishery management measures have been ineffective at maintaining healthy populations. Lalavanua et al. [78] report that the population size structure for this species largely comprises juvenile animals below the size of reproductive maturity, and the general absence of very small individuals of many high-value taxa observed by Jupiter et al. [76] is suggestive of recruitment failure. National management of the sea cucumber fishery in Fiji was formalised through a harvest, processing and export ban on *H*. *scabra* (documented as *Metriatyla scabra*) in 1988, including the export ban of any bêche-de-mer under the size of 3 inches [8, 79]. In 2018 the Fijian Government banned export of all sea cucumber species due to declining populations from mainly visual census surveys [4, 5, 8, 76, 77]. The weak enforcement of the *H*. *scabra* ban, as well as unregulated harvest using highly extractive fishing techniques (e.g., underwater breathing apparatus and free diving with ’bombs’ to access deep water refuges) can be attributed to stock declines [4, 76]. Unfortunately the current outlook on exploitation of all sea cucumber species in Fiji remains grim, with anecdotal reports that the current ban on harvesting may be lifted to allow COVID-19 pandemic relief to coastal communities [80].

Despite the status of the wild sandfish resource in Fiji, data generated by the current study can be used by the Fijian government to formulate national fisheries management plans specifically for this species to permit conservation and recovery of depleted populations. We recommend that special attention be paid to peripheral populations in Lakeba island and Macuata, with dedicated harvesting management or harvesting bans, and monitoring efforts to ensure recovery. It is evident that translocation of individuals (for aquaculture or restocking efforts) from Lakeba island to elsewhere in Fiji not be permitted, given signatures of putative local adaptation. Highly regulated exploitation of the sandfish resource in other areas of Fiji surveyed may be permitted on the basis of genetic data reported here, however a holistic assessment at each site including census population surveys is critical [4, 9, 76] before controlled harvests with comprehensive monitoring can be advised. It is evident that further research on sandfish populations in other areas of Fiji is required to gain a comprehensive understanding of the resource, however the successful use of genome-wide genotyping applied to *H*. *scabra* here can be replicated for other sea cucumber species, to gain insights into the status of the multi-species Fijian sea cucumber fishery as a whole.

#### Restocking efforts

Artificial propagation of juvenile sandfish in hatchery systems offers great promise for potential restoration and recovery of depleted and/or extirpated populations [11], however great care must be taken when selecting broodstock from source populations and the release of hatchery-produced juveniles. It is important that hatchery-based restocking programs ensure adequate levels of genetic diversity are maintained in juveniles and that restocking sites overlap with the natural dispersal limits of the source population [59, 81]. It is imperative that sandfish produced in hatchery systems for restocking are released in close proximity to broodstock source locations, to avoid any ’genetic pollution’ effects arising from the introduction of potentially less diverse individuals into a natural population, or translocation of individuals into a location where natural dispersal regimes may not usually operate. Within the Fiji context, this particularly applies to individuals from Lakeba island, where any hatchery-based restocking efforts will need to utilise locally-sourced broodstock with subsequent release of juveniles in the vicinity of the source location. However, this presents a risk of increasing consanguinity, which may be avoided to an extent by careful genetic selection of broodstock, or controlled reproduction utilising a neighbouring population (e.g. from elsewhere in the Lau archipelago) for introduction of new alleles.

Hatchery production of sandfish juveniles is established in Fiji, through early efforts by Hair et al. [82] and Hair [11]. Since then, the Fiji Ministry of Fisheries regularly carries out hatchery runs to produce juveniles for restocking. One source location for broodstock is Serua island, which reported reduced heterozygosity and an inflated inbreeding coefficient, possibly due to restocking of the natural population with hatchery produced cohorts over several years. Ravago-Gotanco and Kim [59] similarly found reduced effective population size in a wild population they sampled, which was later discovered to have a history of inadvertent restocking with hatchery-produced sandfish.

In other broadcast spawning taxa, losses of genetic diversity have been observed during routine hatchery operations, because of a range of factors including possible ontogenetic changes as a result of interaction with artificial surfaces (e.g., tank walls and algae-coated plates for juvenile settlement) [83], differential fertilisation success [19], differential family and individual larval survival, nutrition and genotype × environment effects [84]. Variability in family survival rates is also possible, where families generated following spawning are lost by the completion of a hatchery run. In the silver-lip pearl oyster, for example, differential family survival was found to range from 2.5–49.5% [19], whereas in barramundi up to 55% of progeny were sired by a single male [84]. The latter scenario may be a concern for sandfish, because although 20–45 broodstock are typically utilised for mass spawning, not all individuals spawn, and female contributions to the larval pool are usually lower [55, 85].

While care must be taken with broodstock selection, spawning management and larval settlement during sandfish hatchery production, considering the particular life history characteristics of this species such as specific settlement/recruitment requirements, an assessment of diversity loss during routine hatchery operations is warranted. Such a study may permit modification of hatchery techniques to minimise differential family survival and even-out broodstock contributions, to ensure production of genetically diverse and unrelated juveniles for replenishment of depleted wild populations.

## Conclusions

This study reports on the genetic diversity, connectivity and structure of Fijian *H*. *scabra* using high resolution genomic data for the first time. It presents directions for the development of fishery management policy for management of both the wild sandfish resource and informs restocking efforts to replenish depleted populations. The successful use of the DArTseq platform in this study has the potential to be applied to other sea cucumber species in Fiji, and in other regions involved in the global bêche-de-mer trade. While this study has offered preliminary insights into the genetic structure and connectivity of sandfish in Fiji, further local, regional and distribution-wide investigations are required to better understand how populations are organised.

## Supporting information

S1 Fig 2012 spawning season simulation El Nino. (GIF)

S2 Fig 2014 spawning season simulation La Nina. (GIF)

S1 File Genotypic data. Genotypes of 211 individuals of *Holothuria scabra* at 6,896 selectively neutral genome-wide SNPs are included in a standard STRUCTURE format.
(XLSX)

S2 File Genotypic data. Genotypes of 211 individuals of *Holothuria scabra* at 186 putatively adaptive genome-wide SNPs are included in a standard STRUCTURE format.
(XLSX)
