# Supplementary figures and images for: Saving the sea cucumbers: Using population genomic tools to inform fishery and conservation management of the Fijian sandfish Holothuria (Metriatyla) scabra
Source: PLoS One. 2022 Sep 9;17(9):e0274245. doi: 10.1371/journal.pone.0274245 (PMC9462726; doi:10.1371/journal.pone.0274245)

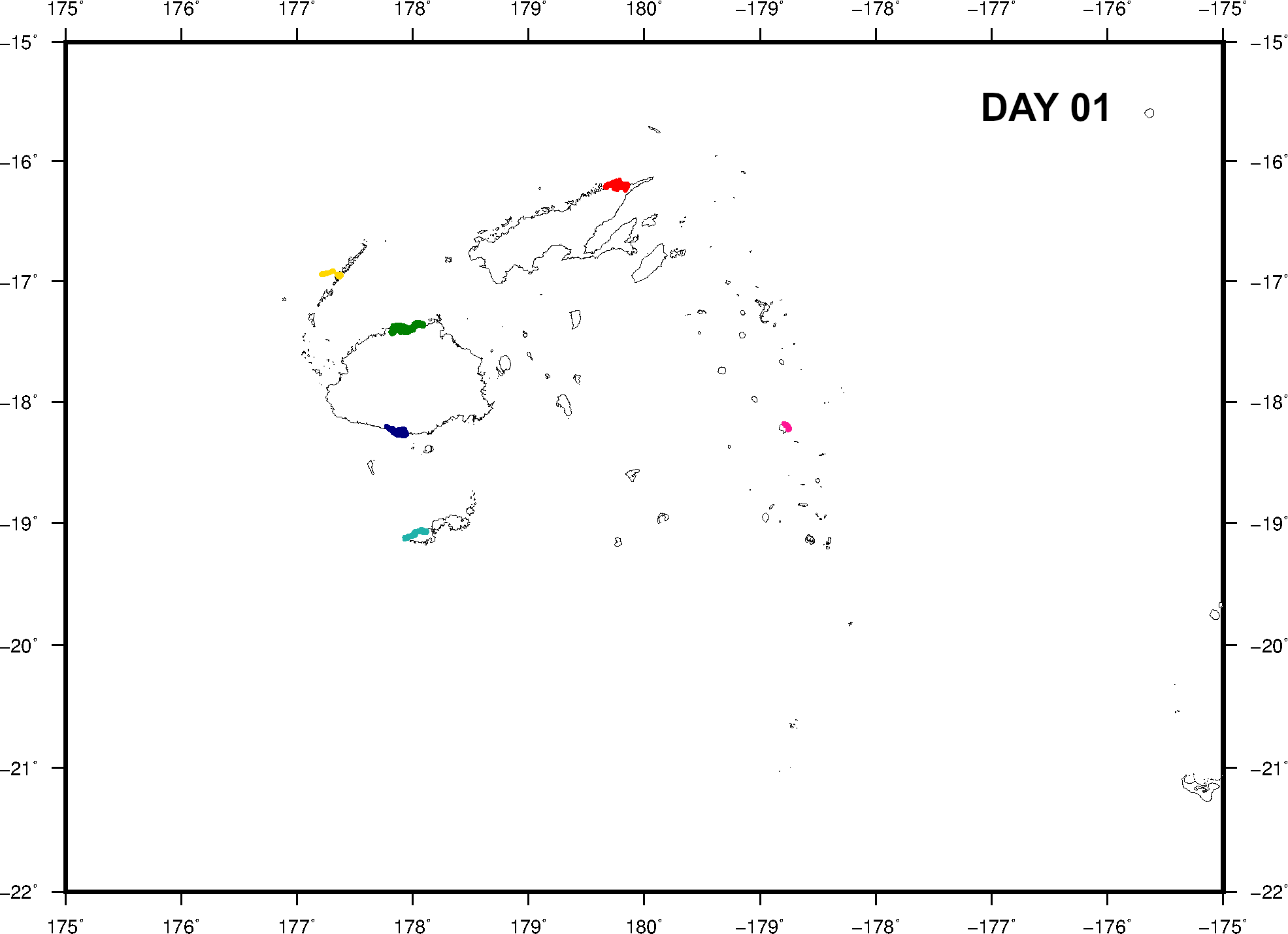

Supplement: S1 Fig — (GIF) [file pone.0274245.s001.gif]

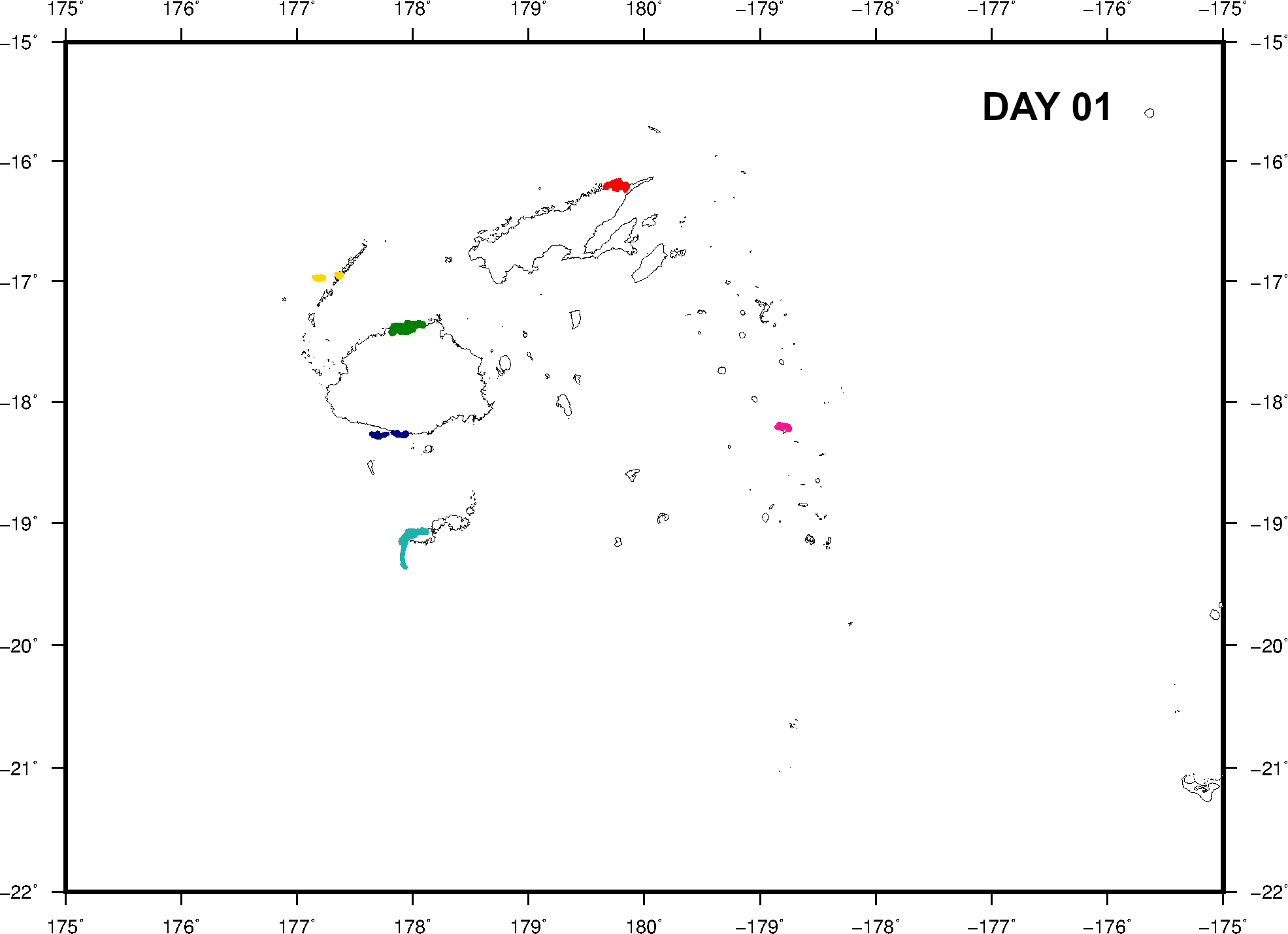

Supplement: S2 Fig — (GIF) [file pone.0274245.s002.gif]
